# Insights into the Mitochondrial Genetic Makeup and Miocene Colonization of Primitive Flatfishes (Pleuronectiformes: Psettodidae) in the East Atlantic and Indo-West Pacific Ocean

**DOI:** 10.3390/biology12101317

**Published:** 2023-10-09

**Authors:** Shantanu Kundu, Flandrianto Sih Palimirmo, Hye-Eun Kang, Ah Ran Kim, Soo Rin Lee, Fantong Zealous Gietbong, Se Hyun Song, Hyun-Woo Kim

**Affiliations:** 1 Institute of Fisheries Science, Pukyong National University, Busan 48513, Republic of Korea; 2 Department of Marine Biology, Pukyong National University, Busan 48513, Republic of Korea; 3 Research Center for Conservation of Marine and Inland Water Resources, National Research and Innovation Agency, Cibinong 16911, Indonesia; 4 Institute of Marine Life Science, Pukyong National University, Busan 48513, Republic of Korea; 5 Marine Integrated Biomedical Technology Center, National Key Research Institutes in Universities, Pukyong National University, Busan 48513, Republic of Korea; 6 The Ministry of Livestock, Fisheries and Animal Industries (MINEPIA), Yaounde 00237, Cameroon; 7 Fisheries Resources Management Division, National Institute of Fisheries Science, Busan 46083, Republic of Korea

**Keywords:** marine fish, ancient lineages, mitogenome, phylogeny, evolution, oceanography

## Abstract

**Simple Summary:**

The present research enriches our comprehension of the mitogenomic genetic features, genetic diversity, evolutionary past, and conservation prerequisites of *Psettodes* flatfishes on a global scale. This study focuses on the matrilineal evolutionary path of these primitive groups, with a specific emphasis on the complete mitogenome of the *Psettodes belcheri* and casting light on its genetic composition, structural traits, and evolutionary chronicle. Exploring genetic variations and phylogenetic relationships uncovers the intricate evolutionary links between *Psettodes* species and their broader context within the Pleuronectiformes species. The complex interplay of hydrographic conditions, ocean currents, and ecological factors emerges as pivotal in shaping the evolutionary landscape of these flatfishes. Given the potential consequences for conservation, this study highlights the necessity for a holistic comprehension of marine environments and the ramifications of climate change and human interventions on flatfish species.

**Abstract:**

The mitogenomic evolution of the *Psettodes* flatfishes is still poorly known from their range distribution in eastern Atlantic and Indo-West Pacific Oceans. The study delves into the matrilineal evolutionary pathway of these primitive flatfishes, with a specific focus on the complete mitogenome of the *Psettodes belcheri* species, as determined through next-generation sequencing. The mitogenome in question spans a length of 16,747 base pairs and comprises a total of 37 genes, including 13 protein-coding genes, 2 ribosomal RNA genes, 22 transfer RNA genes, and a control region. Notably, the mitogenome of *P. belcheri* exhibits a bias towards AT base pairs, with a composition of 54.15%, mirroring a similar bias observed in its close relative, *Psettodes erumei*, which showcases percentages of 53.07% and 53.61%. Most of the protein-coding genes commence with an ATG initiation codon, except for Cytochrome c oxidase I (COI), which initiates with a GTG codon. Additionally, four protein-coding genes commence with a TAA termination codon, while seven others exhibit incomplete termination codons. Furthermore, two protein-coding genes, namely NAD1 and NAD6, terminate with AGG and TAG stop codons, respectively. In the mitogenome of *P. belcheri*, the majority of transfer RNAs demonstrate the classical cloverleaf secondary structures, except for tRNA-serine, which lacks a DHU stem. Comparative analysis of conserved blocks within the control regions of two Psettodidae species unveiled that the CSB-II block extended to a length of 51 base pairs, surpassing the other blocks and encompassing highly variable sites. A comprehensive phylogenetic analysis using mitochondrial genomes (13 concatenated PCGs) categorized various Pleuronectiformes species, highlighting the basal position of the Psettodidae family and showed monophyletic clustering of *Psettodes* species. The approximate divergence time (35−10 MYA) between *P. belcheri* and *P. erumei* was estimated, providing insights into their separation and colonization during the early Miocene. The TimeTree analysis also estimated the divergence of two suborders, Psettodoidei and Pleuronectoidei, during the late Paleocene to early Eocene (56.87 MYA). The distribution patterns of *Psettodes* flatfishes were influenced by ocean currents and environmental conditions, contributing to their ecological speciation. In the face of climate change and anthropogenic activities, the conservation implications of *Psettodes* flatfishes are emphasized, underscoring the need for regulated harvesting and adaptive management strategies to ensure their survival in changing marine ecosystems. Overall, this study contributes to understanding the evolutionary history, genetic diversity, and conservation needs of *Psettodes* flatfishes globally. However, the multifaceted exploration of mitogenome and larger-scale genomic data of *Psettodes* flatfish will provide invaluable insights into their genetic characterization, evolutionary history, environmental adaptation, and conservation in the eastern Atlantic and Indo-West Pacific Oceans.

## 1. Introduction

Flatfishes, also known as flounders, soles, halibuts, turbots, plaices, and tonguefishes, are classified as Pleuronectiformes. This order is split into two suborders: Psettodoidei, which includes the single-family Psettodidae, and Pleuronectoidei, which includes 13 families [1]. Flatfishes live mostly in tropical and subtropical marine habitats, preferring shallow parts of the continental shelf with soft sandy bottoms [2]. Their evolutionary journey, particularly marked by cranial asymmetry and unique scale structures, has facilitated their successful adaptation and dominance in benthic aquatic habitats [3,4,5]. The genus *Psettodes* is a “primitive” flatfish group within the monotypic Psettodidae family, with three recognized species: *Psettodes belcheri*, *Psettodes bennettii*, and *Psettodes erumei* [6]. While *P. erumei*, commonly referred to as the Indian halibut, ranges expansively across the Red Sea and the Indo-West Pacific Ocean, the other two species are confined to the eastern Atlantic. Intriguingly, the ranges of *P. belcheri* and *P. bennettii* exhibit partial overlap, spanning from the west Sahara to the Liberian coast.

The economic significance of flatfishes has led to their frequent capture through demersal trawling in marine ecosystems [7]. The Indian halibut (*P. erumei*) is heavily harvested within the tropical fishing zone as defined by the United Nations Food and Agriculture Organization (FAO) [8]. Despite this heavy exploitation, the IUCN Red List classifies information on the status of these three *Psettodes* species as ‘Data Deficient.’ [9]. Furthermore, the extraordinary transformation from bilateral pelagic larval symmetry to adult flattened symmetry limits flatfishes to demersal zones on the sea bottom, owing to their unusual temperature-mediated spawning behavior [10]. While most flatfish species have either a right-sided (dextral) or a left-sided (sinistral) mouth, several species, including *Psettodes*, display varying degrees of dextral to sinistral polymorphism [11]. This sexual dimorphism in external morphology often poses challenges in species identification. Moreover, this characteristic complexity in external appearance frequently hinders precise species differentiation among flatfishes [12].

The integration of molecular data has become integral in the exploration of global flatfish species, encompassing diverse aspects such as species identification [13], systematic classification [14], phylogenetic relationships [15], and population structure estimation [16]. Genetic information has also found utility in identifying valuable commercial species and managing aquaculture stocks [17,18]. The acquisition of comprehensive genomic resources is pivotal for advancing flatfish research and its manifold applications [19]. While the origins of flatfishes have long been debated, recent molecular insights lend support to the concept of a ‘lower-percoid’ origin, a perspective gaining prominence in the field of teleost ichthyology [20,21,22]. The taxonomic placement of *Psettodes* has stirred argument, especially in the context of analyses involving nuclear and mitogenomic data. This genus consistently diverges from other flatfishes (Pleuronectiformes), instead aligning with other carangimorphs [23,24,25,26]. A comprehensive phylogenetic analysis utilizing 1000 ultraconserved DNA element loci across 45 carangimorphs subsequently solidified the monophyly of flatfishes [27]. Later studies, involving complete mitogenome-based phylogeny and molecular clock analyses, offered insights into the enigmatic placement and evolutionary divergence of the *Psettodes* genus within the context of other carangimorphs and pleuronectoids [28,29]. However, these investigations were limited to a single *Psettodes* taxon (*P. erumei*) from the Indo-Pacific region, highlighting the need for expanded inquiries involving other congeners from the Atlantic Ocean.

An integrated strategy that looks beyond superficial investigation is required for an accurate appraisal of speciation within maritime ecosystems. Advances in technology, spanning maritime engineering to genomics, are facilitating the investigation of marine speciation [30,31]. To enhance our grasp of *Psettodes’* maternal evolutionary path, this study seeks to construct the complete mitogenome of *P. belcheri*, elucidate its genomic attributes, and establish its phylogenetic relationships. This endeavor contributes to understanding the maternal evolution of these ancient lineages in marine environments, strengthening the mitogenomic repository of flatfishes, and expanding our scientific insight into the Psettodidae family on a global scale. While marine habitats harbor a diverse array of life forms, our understanding of speciation in marine ecosystems is comparatively limited compared with freshwater systems, necessitating urgent exploration of the mechanisms driving speciation and adaptation [32]. Given the unique distribution of Psettodidae flatfishes across the eastern Atlantic, Red Sea, and Indo-West Pacific Ocean, this study also aims to estimate the divergence time between two Psettodidae species (*P. belcheri* and *P. erumei*) and investigate potential evolutionary scenarios within the marine environment.

## 2. Materials and Methods

### 2.1. Sampling and Species Identification

**Figure 1:**
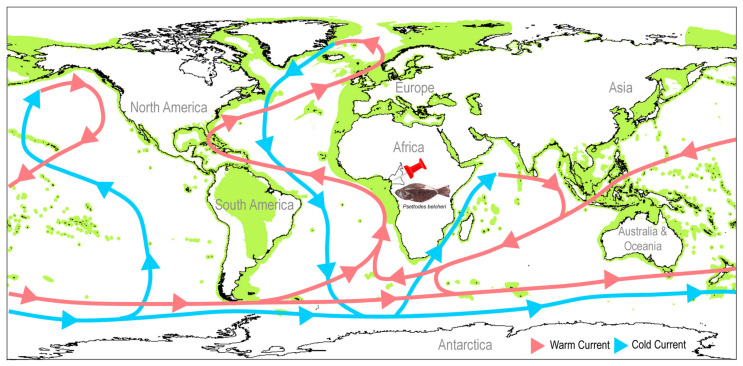
Global distribution of Pleuronectiformes along with the ocean surface currents (warm and cold currents are marked by the red and blue arrow, respectively). Collection locality of *Psettodes belcheri* is marked by a red pin from Cameroon, Africa. The .shp files were acquired from the IUCN database (accessed on 15 August 2023). The species photographs were taken by the sixth author (F.Z.G) and edited manually in Adobe Photoshop CS 8.0.

A solitary specimen of the spottail spiny turbot, scientifically known as *P. belcheri*, was acquired from the estuaries of the Kineke River (latitude 2.938611° N, longitude 9.911667° E) located in Kribi, Cameroon (Figure 1). The identification process was meticulously carried out in accordance with available taxonomic keys [33]. Upon euthanizing the specimen with MS-222 (200 mg/L), muscle tissue was aseptically collected from the ventral thoracic region. The voucher specimen was duly archived in 10% formaldehyde at the Fisheries and Animal Industries department (MINEPIA) in Yaoundé, Cameroon. Simultaneously, a tissue sample was preserved at the Department of Marine Biology, Pukyong National University in Busan, South Korea. The Institutional Animal Care and Use Committee of the host institute granted approval (Approval Code: PKNUIACUC-2022-72, dated 16 December 2022) for the utilization of deceased fish muscle tissue in molecular investigations. To enhance our comprehension of their geographical distribution, global maps of Pleuronectiformes, inclusive of the range distributions of *P. belcheri* and its congeners (*P. erumei* and *P. bennettii*), were obtained in .shp file format from the IUCN database (https://www.iucnredlist.org/, accessed on 15 August 2023) (Figure 1).

### 2.2. DNA Extraction, Mitogenome Sequencing, and Assembly

The AccuPrep^®^ Genomic DNA extraction kit, manufactured by Bioneer in Daejeon, Republic of Korea, was utilized to extract genomic DNA, following the established standard protocol. The quality and quantity of the resulting genomic DNA were meticulously evaluated employing a NanoDrop spectrophotometer (Thermo Fisher Scientific D1000, WA, USA). To obtain the comprehensive mitogenome of *P. belcheri*, sequencing procedures were executed using the NovaSeq platform, provided by Illumina and accessible at Macrogen (https://dna.macrogen.com/, accessed on 15 August 2023) in Daejeon, Republic of Korea. The sequencing libraries were prepared following the manufacturer’s instructions for the TruSeq Nano DNA High-Throughput Library Prep Kit (Illumina, Inc., San Diego, CA, USA). In short, 100 ng of genomic DNA underwent fragmentation using adaptive focused acoustic technology (Covaris, Woburn, MA, USA), resulting in blunt-ended dsDNA molecules with 5′-phosphorylation. After the end-repair step, DNA fragments were size selected using a bead-based method. These fragments were then modified with the addition of a single ‘A’ base and ligated with TruSeq DNA UD Indexing adapters. Subsequently, the products were purified and enriched through PCR to create the final DNA library. Library quantification was performed using qPCR, following the qPCR Quantification Protocol Guide (KAPA Library Quantification kits for Illumina Sequencing platforms), and quality assessment was carried out using Agilent Technologies 4200 TapeStation D1000 screentape (Agilent Technologies, Santa Clara, CA, USA). Finally, paired-end (2 × 150 bp) sequencing was performed by Macrogen using the NovaSeq platform (Illumina, Inc., San Diego, CA, USA). Over 20 million raw reads underwent processing using the Cutadapt tool (http://code.google.com/p/cutadapt/, accessed on 15 August 2023) to trim adapters and remove low-quality bases, with a Phred quality score (Q score) cutoff of 20. The Geneious Prime version 2023.0.1 was used to assemble the targeted genome from the high-quality paired-end NGS reads. This assembly was accomplished by employing reference mapping with the mitogenome of a closely related species as a reference, and we employed default mapping algorithms. Mitogenome assembly was accomplished by scrutinizing the alignment of overlapping regions via MEGA X [34]. The boundaries and orientations of individual genes were validated using the MITOS v806 (http://mitos.bioinf.uni-leipzig.de, accessed on 15 August 2023) and MitoAnnotator (http://mitofish.aori.u-tokyo.ac.jp/annotation/input/, accessed on 15 August 2023) web servers [35,36]. To further corroborate protein-coding genes (PCGs), the translated putative amino acid sequences were scrutinized using the Open Reading Frame Finder web tool (https://www.ncbi.nlm.nih.gov/orffinder/, accessed on 15 August 2023), based on the vertebrate mitochondrial genetic code. The resultant mitogenome of *P. belcheri* was duly submitted to the global GenBank database.

### 2.3. Mitogenomic Characterization

A spherical representation of the generated mitogenome was crafted using MitoAnnotator (http://mitofish.aori.u-tokyo.ac.jp/annotation/input/, accessed on 15 August 2023). A comprehensive comparative analysis was carried out to assess the mitogenomic architecture and variations within our generated sequence in relation to two pre-existing mitogenomes from a single congener, *P. erumei* (FJ606835, sourced from China, and AP006835, sourced from Japan). The calculation of intergenic spacers, which separate adjacent genes, and overlapping regions was performed manually. To determine the nucleotide compositions of protein-coding genes (PCGs), ribosomal RNA (rRNA), transfer RNA (tRNA), and the control region (CR), we employed MEGA X [34]. Similarly, base composition skews, as previously detailed, were computed using the following formulas: AT-skew = [A − T]/[A + T], GC-skew = [G − C]/[G + C] [37]. The verification of initiation and termination codons for each PCG, as well as adherence to the vertebrate mitochondrial genetic code, was carried out using MEGA X. Additionally, the boundaries of rRNA and tRNA genes were confirmed through the use of the tRNAscan-SE Search Server 2.0 in conjunction with ARWEN 1.2 [38,39]. Structural domains within the control region were delineated through CLUSTAL X alignments [40], and tandem repeats were explored utilizing the online Tandem Repeats Finder web tool (https://tandem.bu.edu/trf/trf.html, accessed on 15 August 2023) [41].

### 2.4. Genetic Distance, Phylogenetic Analyses, and TimeTree Estimation

Genetic distances were computed using the Kimura 2-parameter (K2P) method within MEGA X. Due to the unavailability of the complete mitogenome of *P. bennettii*, intra-species and inter-species distances were determined using the widely employed mitochondrial COI gene. To elucidate the matrilineal phylogenetic connections, 14 mitogenomes from 13 Pleuronectiformes species were sourced from the GenBank database (accessed on 15 August 2023) [28,42,43,44,45,46,47,48,49] (Table S1). Dataset preparation adhered to methodologies outlined in two recent Pleuronectiformes studies [26,50]. The spotfin flounder, *Cyclopsetta fimbriata*, was categorized within the recently established family Cyclopsettidae (=Paralichthyidae II). Additionally, two Cynoglossidae species were included: *Cynoglossus gracilis* (=Cynoglossidae I) and *Symphurus orientalis* (=Cynoglossidae II). The mitogenome of *Lates calcarifer* (DQ010541), from the Centropomidae family, was incorporated as an outgroup. Concatenation of all 13 PCGs was executed using the iTaxoTools 0.1 tool to construct the dataset for phylogenetic analysis [51]. In order to prevent inadvertent gaps within the dataset alignment, we consciously excluded the non-coding rRNA genes and control regions from the current phylogenetic analysis. Model selection yielded the ‘GTR + G + I’ model as the most suitable, determined by the lowest Bayesian Information Criterion (BIC) score using PartitionFinder 2 through CIPRES Science Gateway v3.3 and JModelTest v2 [52,53,54]. Employing Mr. Bayes 3.1.2, a Bayesian (BA) tree was constructed, employing nst = 6, along with one cold and three hot Metropolis-coupled Markov Chain Monte Carlo (MCMC) chains. The analysis spanned 10,000,000 generations with tree sampling at every 100th generation and 25% of samples discarded as burn in [55]. Visualization of the BA tree was accomplished using the iTOL v4 web server (https://itol.embl.de/login.cgi, accessed on 15 August 2023) [56]. Additionally, the divergence time estimation was performed using the RelTime method following standard protocol as implemented in MEGA X [57]. This approach was intended to reduce the large computational time involved in Bayesian methods [58,59]. After loading the sequences data, the constructed maximum-likelihood topology (.nwk format) was used as a baseline tree. After specifying the outgroup taxa, the TimeTree computation incorporated two calibration constraints through the calibration editor: the divergence from the sister lineage of Citharidae (55.54 MYA) and Achiridae (49.73 MYA), as established in a previous study [28].

## 3. Results and Discussion

### 3.1. Mitogenomic Structure and Organization

**Figure 2:**
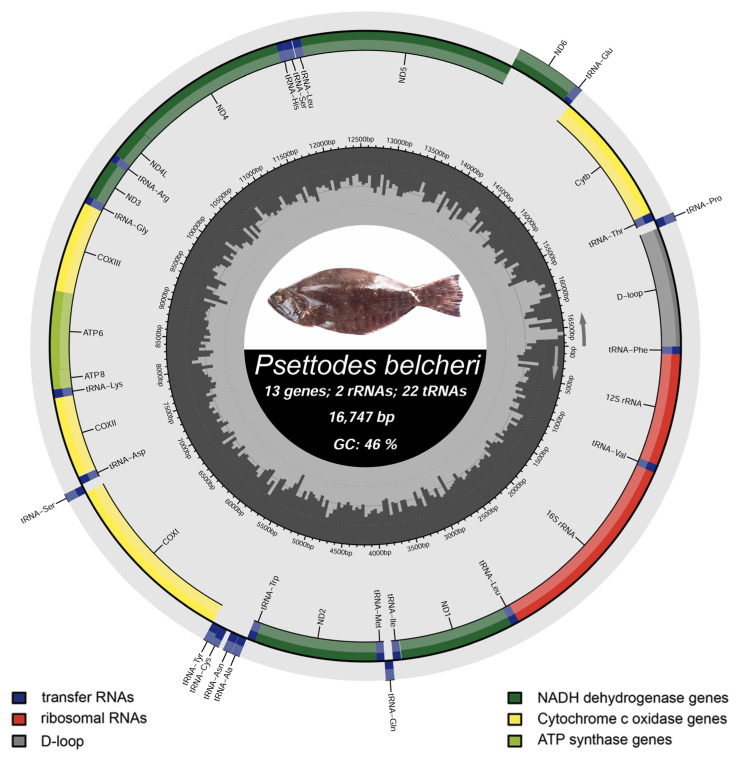
The mitochondrial genome of *Psettodes belcheri* drawn by the MitoAnnotator online server. PCGs, rRNAs, tRNAs, and CR are indicated by different color arcs. The species photographs were taken by the sixth author (F.Z.G) and edited manually in Adobe Photoshop CS 8.0.

In this study, the mitogenome of *P. belcheri* (16,739 bp) was characterized and assigned the GenBank accession number OR231239. The circular mitogenome of *P. belcheri* comprised 13 protein-coding genes (PCGs), 22 transfer RNA genes (tRNAs), 2 ribosomal RNA genes (rRNAs), and a non-coding AT-rich control region (CR). Among these, 28 genes (12 PCGs, 2 rRNAs, and 14 tRNAs) were positioned on the heavy strand, while NAD6 and 8 tRNAs (trnQ, trnA, trnN, trnC, trnY, trnS2, trnE, and trnP) were situated on the light strand (Table 1, Figure 2).

A similar distribution of genes on heavy and light strands was evident in other Pleuronectiformes species, with total lengths ranging from 16,506 bp (*C. fimbriata*, family Cyclopsettidae, AP014590) to 18,706 bp (*Samariscus latus*, family Samaridae, KF494223). Variability in mitogenomic lengths was largely attributed to duplications in the control regions (CRs) [44]. The mitogenome of *P. belcheri* showcased five overlapping regions, spanning a total of 14 bp. The longest overlap (7 bp) was observed between ATP synthase 8 (atp8) and ATP synthase 6 (atp6). Similarly, both *P. erumei* mitogenomes (FJ606835 and AP006835) displayed five overlapping regions totaling 23 bp, with the longest overlap (10 bp) between atp8 and atp6, mirroring *P. belcheri*. Moreover, *P. belcheri* exhibited 15 intergenic spacer regions spanning a total of 106 bp, with the longest (38 bp) situated between trnN and trnC. Conversely, *P. erumei* mitogenomes contained 11 intergenic spacer regions spanning 77–78 bp, with the longest (37–38 bp) found between trnN and trnC (Table S2). Nucleotide composition analysis revealed the AT-biased nature of *P. belcheri* mitogenome (54.15%), encompassing 28.09% A, 16.24% G, 29.56% C, and 26.06% T. Similar AT richness was observed in the nucleotide composition of the other two *P. erumei* mitogenomes, ranging from 53.07% to 53.61% (Table 2). The AT skew and GC skew of *P. belcheri* mitogenome were recorded as 0.037 and −0.291, respectively. Comparative analysis of *P. erumei* mitogenomes demonstrated an AT skew range of 0.076 to 0.077, and a GC skew range of −0.323 to −0.328 (Table 2). This consistent nucleotide composition pattern and AT bias were also observed in other previously described fish mitogenomes [60,61]. The genetic variations identified within the *Psettodes* mitogenome may be tied to their evolutionary progression and energy metabolism, echoing findings in other fish species [62]. This research offers valuable insights into the structural attributes of *Psettodes* mitogenomes, which hold significance in deciphering the functionalities of these mitogenomes and their encoded genes.

### 3.2. Protein-Coding Genes

A cumulative length of 11,427 bp in *P. belcheri* mitogenome was occupied by a total of 13 protein-coding genes (PCGs), accounting for 68.27% of the whole sequence. Among these, the shortest PCG was ATP8, spanning 165 bp, while NAD5 represented the longest PCG with a length of 1839 bp. In both *P. erumei* mitogenomes, the total length of PCGs ranged from 11,426 bp (68.49%) to 11,427 bp (65.99%). The PCGs of *P. belcheri* were characterized by an AT bias of 52.73%, accompanied by AT skew and GC skew values of −0.052 and −0.323, respectively (Table 2). Similarly, the mitogenomes of *P. erumei* displayed an AT bias ranging from 50.95% to 51.12%, coupled with AT skew values of −0.008 to −0.006 and GC skew values of −0.363 to −0.361. Most of the PCGs commenced with an ATG (Methionine) initiation codon, except for COI, which began with a GTG (Valine) codon. A parallel pattern of initiation codons was apparent in all PCGs of the other two *P. erumei* mitogenomes. Among the PCGs, the conventional TAA termination codon was observed in four instances (COI, ATP8, NAD4L, and NAD5), while seven PCGs featured incomplete stop codons (T--/TA-). Additionally, two PCGs, NAD1 and NAD6, terminated with AGG and TAG stop codons, respectively. A corresponding distribution of stop codons was also noted in *P. erumei* mitogenomes (Table 2 and Table S3). These incomplete stop codons could potentially be completed with TAA during RNA processing, as previously suggested [63]. As observed in other fish species, the identified genetic disparities might lead to the independent selection of PCGs [64]. PCGs play pivotal roles in oxidative phosphorylation, ATP synthesis, and the encoding of proteins within the electron transport pathways. Consequently, the inclusion of mitogenomes from various *Psettodes* species could facilitate the exploration of variations in gene expression and energy utilization.

### 3.3. Ribosomal RNA and Transfer RNA Genes

**Figure 3:**
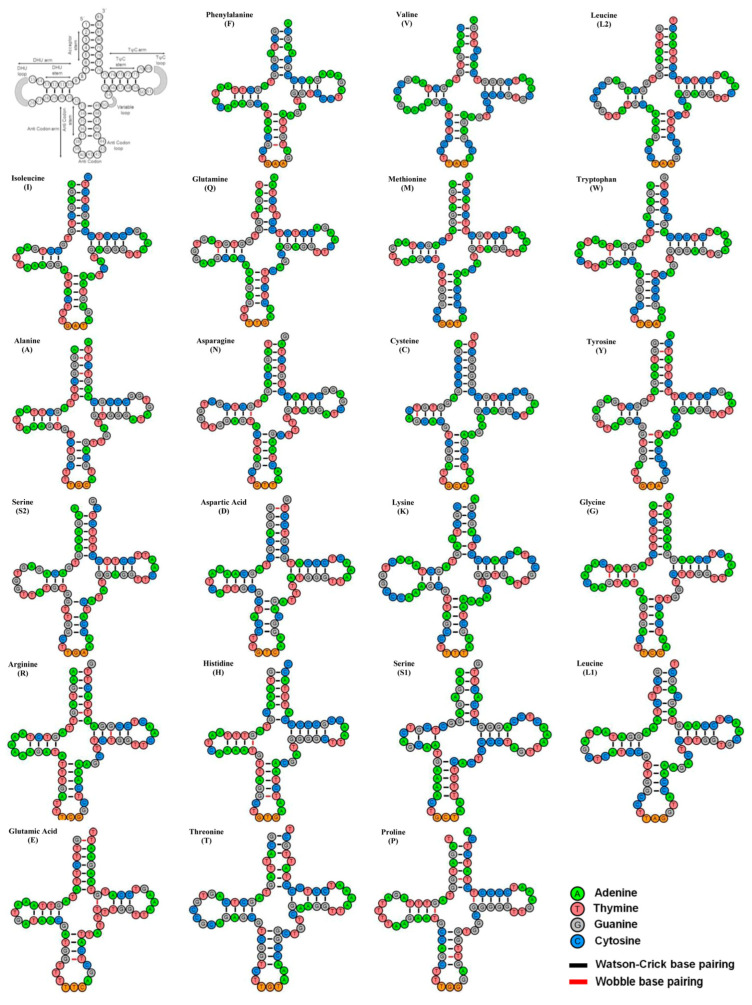
Secondary structures of 22 transfer RNAs (tRNAs) of *P. belcheri* display the structural variation. The tRNAs are denoted by full names and IUPAC-IUB single-letter amino acid codes. The first structure shows the nucleotide positions and details of the stem–loop of tRNAs. Watson–Crick and wobble base pairing are marked by black and red color bars, respectively.

Within the *P. belcheri* mitogenome, the ribosomal RNA genes collectively spanned 2689 bp, equivalent to 16.06% of the complete mitogenome. This encompassed a small ribosomal RNA (12S rRNA) measuring 961 bp and a large ribosomal RNA (16S rRNA) with a length of 1702 bp. Comparatively, the total length of *P. erumei*’s rRNAs (2680 bp) was shorter than that of *P. belcheri* (Table 2). The ribosomal RNAs AT composition ranged from 52.81% (*P. belcheri*) to 52.99% (*P. erumei*). Comparative analysis indicated AT skew values ranging from 0.190 (*P. belcheri*) to 0.227 (*P. erumei*), while GC skew values ranged from −0.113 (*P. erumei*) to −0.073 (*P. belcheri*) in the ribosomal RNA (Table 2). These rRNA genes’ structural arrangement, notably the conserved loops, offer critical insights into the catalytic chemical processes underlying protein synthesis [65]. Additionally, the *P. belcheri* mitogenome featured 22 tRNA genes, exhibiting varying lengths from 66 bp (trnC) to 75 bp (trnK), collectively constituting 1556 bp or 9.29% of the entire mitogenome. The combined tRNA length in both *P. erumei* mitogenomes was shorter (1553 bp and 1554 bp) than that of *P. belcheri* (Table 2). The tRNAs displayed an AT bias in both *P. belcheri* (55.14%) and *P. erumei* (54.02% to 54.12%). AT skew values ranged from 0.006 (*P. erumei*) to 0.009 (*P. belcheri*), while GC skew values ranged from 0.032 (*P. belcheri* and *P. erumei*, AP006835) to 0.036 (*P. erumei*, FJ606835) (Table 2). Most tRNAs were predicted to adopt the typical cloverleaf secondary structure, except for trnS1, which lacked the DHU stem, consistent with findings in other Pleuronectiformes [66,67]. These genetic attributes are pivotal for shaping transfer RNAs secondary structures and their functional roles within diverse biological systems [68]. In terms of comparative structural features, 15 tRNA genes (trnA, trnF, trnQ, trnM, trnW, trnN, trnC, trnY, trnS2, trnD, trnG, trnR, trnH, trnE, trnP) were constructed through both conventional Watson–Crick base (A=T and G≡C) pairing and wobble base pairing (G-T), while the remaining seven tRNA genes were exclusively built with Watson–Crick base pairs (Figure 3).

### 3.4. Features of Control Region and Gene Arrangements

**Figure 4:**
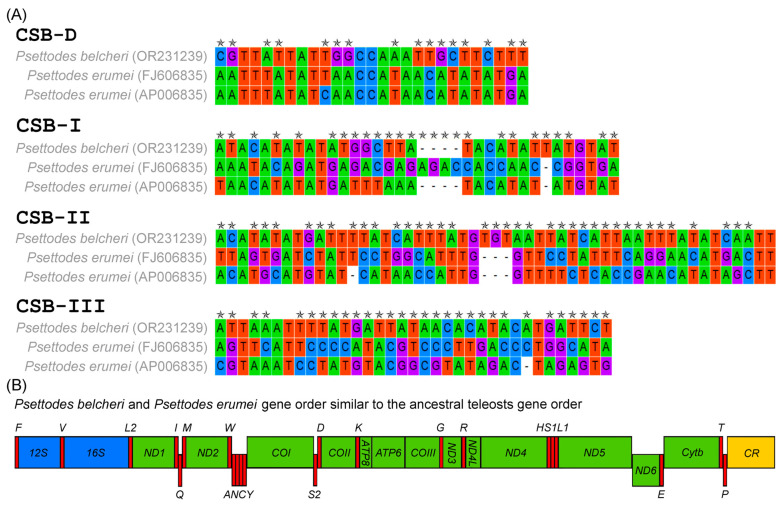
(**A**) Comparison of length and nucleotide composition of four conserved domains of two *Psettodes* species control regions. The variable nucleotides are marked in stars; (**B**) The gene arrangements of two *Psettodes* species mitogenomes.

The comprehensive length of *P. belcheri* control region (CR) reached 1015 bp, comprising 63.65% AT content. Conversely, the complete lengths of *P. erumei*’s CRs varied, ranging from 968 bp to 1601 bp. The AT skew fluctuated between −0.050 (*P. belcheri*) and 0.074 (*P. erumei*), while the GC skew ranged from −0.337 (*P. erumei*, AP006835) to −0.241 (*P. erumei*, FJ606835) (Table 2). *P. belcheri* mitogenome recorded more than two copies of 72 bp tandem repeats, whereas the mitogenomes of *P. erumei* hosted over eight copies (FJ606835) and more than twelve copies (AP006835) of 56 bp repeats. Four conserved blocks (CSB-D, CSB-I, CSB-II, and CSB-III) were detected in both *P. belcheri* and *P. erumei* mitogenomes, consistent with their presence in other teleost fishes [66,69]. Of these blocks, CSB-II was the longest at 51 bp, compared with CSB-D (27 bp), CSB-I (36 bp), and CSB-III (35 bp) (Figure 4A). Comparative analyses unveiled substantial nucleotide variability and parsimony informative nucleotides within CSB-II relative to the other three conserved domains. This AT-rich regulatory region holds the potential for assessing population structures and identifying inter- and intra-specific differences among *Psettodes* species via these variable nucleotides. As demonstrated in other species, such conserved domains are integral for mitochondrial genome replication and transcription [69,70]. Intriguingly, these primitive Pleuronectiformes fishes maintain a gene order within their mitochondrial genomes that aligns with that of ancestral teleosts [71] (Figure 4B). However, Pleuronectiformes mitogenomes exhibit repeated instances of control region duplications and gene rearrangements [72,73,74,75,76]. These mechanisms involving genomic rearrangement through double replications, random loss, dimer-mitogenomes, and non-random loss contribute to understanding the structural diversity of mitogenomes and the intricacies of mitochondrial genome evolution.

### 3.5. Genetic Distances and Mitogenomic Phylogeny

**Figure 5:**
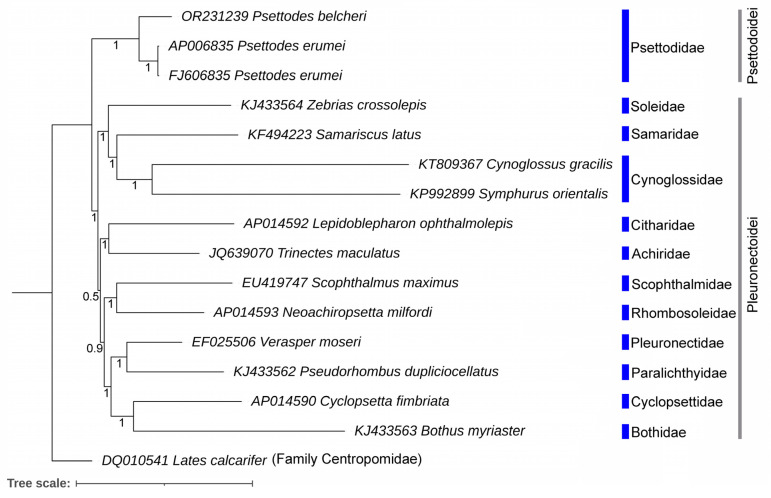
The Bayesian matrilineal phylogeny based on the concatenated sequences of 13 PCGs exhibits the evolutionary relationship of *Psettodes* species with other Pleuronectiformes. The Bayesian posterior probability values are displayed with each node.

The species under investigation, *P. belcheri*, displayed substantial inter-species genetic distances of 14.00% and 17.30% when compared with its congeners *P. bennettii* and *P. erumei*, respectively, as confirmed through the analysis of the COI gene (Figure S1). A particularly noteworthy finding is the pronounced genetic divergence of 14% observed between *P. belcheri* and *P. bennettii*, despite their shared presence in the western Atlantic. This genetic disparity, noteworthy in the context of their parapatric distribution and significant overlap, points to a significant level of reproductive isolation between these species. The examination of population genetic structures within *P. belcheri* and *P. bennettii* will provide valuable insights into their migratory patterns within the mutual habitat of the eastern Atlantic Ocean. Employing a phylogenetic analysis grounded in mitogenomes, the study effectively categorized all studied Pleuronectiformes flatfishes using a concatenation of 13 PCGs, supported by robust posterior probability (Figure 5). The resultant phylogeny derived from mitogenomes aligns harmoniously with previous evolutionary hypotheses concerning Pleuronectiformes species [26,28,29]. Importantly, representative species from the suborders Psettodoidei and Pleuronectoidei formed coherent monophyletic clusters within the present topology. The spottail spiny turbot, *P. belcheri*, clustered consistently with its congeners, most notably *P. erumei*. The family Psettodidae emerged as the basal node of Pleuronectiformes, occupying a unique position as an ancestral group among other flatfish families. Furthermore, the conducted cladistic analysis showcased a sister relationship between *C. gracilis* (=Cynoglossidae I) and *S. orientalis* (=Cynoglossidae II) (Figure 5). The application of mitochondrial genome-based and phylogenomic assessments has proven successful in elucidating higher teleostean phylogenies, encompassing flatfishes [77,78,79]. To establish the precise matrilineal evolution of *Psettodes* flatfishes within the monotypic family Psettodidae, the generation of the *P. bennettii* mitogenome emerges as a pivotal task. Notably, a wealth of genetic data, spanning multi-locus exon-capture data and whole genome sequencing, has recently provided fresh insights into the phylogeny and genetic evolution of flatfishes [80,81,82,83,84,85]. The integration of such extensive genetic information depicts the potential to illuminate the evolutionary landscape of primitive *Psettodes* flatfishes in the near future.

### 3.6. Divergence Time and Diversification

**Figure 6:**
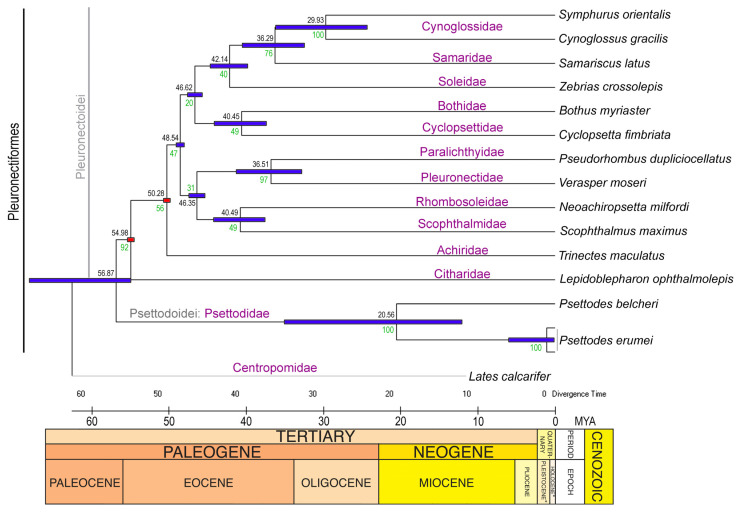
The maximum-likelihood-based TimeTree elucidates the approximate divergence time of the *Psettodes* species along with other *Pleuronectiformes* species. The approximate divergence times are marked displayed with each node. The red squares represent the calibration points obtained from the prior work [26].

The application of TimeTree analyses has illuminated the temporal aspects of the evolutionary history of *P. belcheri* and *P. erumei*. These two species have a wide divergence time during the late Eocene to early Miocene, approximately 35 to 10 million years ago (MYA) (Figure 6). Remarkably, the family Psettodidae (suborder Psettodoidei) demonstrated an early divergence of approximately 56.87 MYA from other flatfish families (suborder Pleuronectoidei) during the late Paleocene to early Eocene (Figure 6). The other Pleuronectoidei flatfish families diverged from each other between the Oligocene and early Eocene periods, with dates ranging from 29.93 MYA to 54.98 MYA (Figure 6). The distribution pattern of *Psettodes* flatfishes, specifically within the family Psettodidae, has fascinated many ichthyologists. The interannual variability in early life phenology and dispersal of flatfishes has been established as being influenced by bathymetry, changes in water salinity, oceanic temperature, and wind conditions [86,87]. Thus, the study of the evolution and diversification of marine fishes necessitates a discussion that takes into account genetic connectivity, divergent selection, as well as possible demographic and ecological opportunities [88].

The Miocene epoch, spanning from around 23 million to 5.3 million years ago, marked a period of substantial environmental transformation on Earth [89]. This era witnessed shifts in both climate and oceanic conditions, representing a transition between the stable warmth of the early Cenozoic and the later variable and colder conditions [90]. During the Miocene, elevated temperatures contributed to higher sea levels through the melting of polar ice and seawater expansion [91]. Furthermore, the tectonic activity in the Miocene shaped ocean basins and seafloor topography, with the collision of tectonic plates, influencing ocean currents and marine habitats. Consequently, coastlines took on different configurations than those seen today, with numerous present-day land areas submerged under shallow seas. The epoch was characterized by significant biological evolution, with the emergence of contemporary marine species lineages influencing the rich biodiversity of modern oceans [92,93]. Such diversification was prompted by factors such as varying oceanic temperatures, changing sea levels, and the availability of ecological niches.

**Figure 7:**
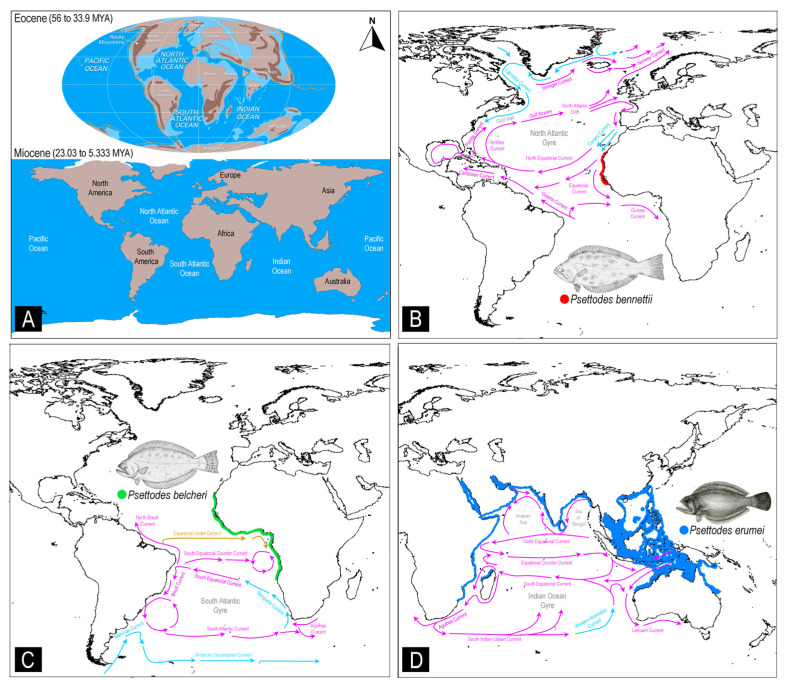
(**A**) Maps displaying the maritime environments during the Eocene and Miocene periods (source: Encyclopædia Britannica, Inc.). Schematic representation of the major current systems, as defined by the International Group for Marine Ecological Time Series, in the North Atlantic, South Atlantic, and Indo-West Pacific regions. This section also explores the potential diversification and colonization of primitive flatfishes: (**B**) *P. bennettii*, (**C**) *P. belcheri*, and (**D**) *P. erumei*. The illustration of the *Psettodes* species was sourced from the free media repository (Wikimedia Commons), as well as a previous study [33]. The maps were generated using the DIVA-GIS platform, utilizing IUCN range distribution data (.shp files). Additionally, the illustration of ocean currents is based on insights from a previous study [94]. Violet arrows indicate the warmer currents, while the blue arrows signify the cooler currents.

In the context of understanding the evolutionary scenarios of *Psettodes* flatfishes, particularly following their colonization in the eastern Atlantic and Indo-West Pacific regions by the demersal lineage, a critical integration of the maximum-likelihood time-tree computation framework and marine ecological factors becomes essential. Notably, while *P. belcheri* is distributed across both the South and North Atlantic Oceans, spanning the western coast of Africa from Angola to western Sahara, *P. bennettii* is confined solely to the North Atlantic, with a distribution encompassing Gambia, Guinea, Guinea-Bissau, Mauritania, Morocco, Senegal, and Western Sahara. The sympatric speciation of these two species may be attributed to ecological selection within the pelagic environment. Since ancient time, the hydrographic and climatic conditions in the North and South Atlantic Oceans have differed significantly due to the Coriolis effect induced by Earth’s rotation (Figure 7A). In the North Atlantic, the circulation of oceanic currents results in distinct oceanic gyres, with the warm Gulf Stream current flowing northward and the cold Canary Current flowing southward [94]. The interplay of these currents, along with the North Equatorial Current, contributes to the formation of the North Atlantic gyre (Figure 7B). Conversely, the southern Atlantic Ocean features counterclockwise current circulation, dominated by the anticyclonic subtropical gyre and bounded by several major surface ocean currents (the Antarctic Circumpolar Current, the Benguela Current, the South Equatorial Current, the Brazil Current, and the Malvinas Current) (Figure 7C).

The Indian Ocean, characterized by the presence of the Arabian Sea and the Bay of Bengal, as well as warm and cold currents such as the Western Australian cold current, forms the Indian Ocean gyre. The divergence time of Psettodidae from other Pleuronectiformes during the late Paleocene to early Eocene suggests that these primitive flatfishes may have emerged in the eastern Atlantic Ocean. The currents and salinity levels of both the North and South Atlantic Oceans may have constrained the distribution of the two Psettodidae species, *P. belcheri* and *P. bennettii*, to the continental shelves of western Africa. The other congener, *P. erumei*, likely evolved during the early Miocene and colonized into the Red Sea to the Indo-West Pacific Ocean due to the formation of the Antarctic Circumpolar Current (Figure 7D). Notably, the Agulhas Current may have acted as a barrier to the distribution of *P. erumei* between the Indian Ocean and the Atlantic Ocean. Despite the differences in hydrography, the high saline outflow from the Red Sea to the Indian Ocean results in similar characteristics of these two marine environments, allowing for similar flatfish communities to thrive. However, the Indian Ocean and Western Pacific Ocean are seismically active due to the presence of tectonic plate boundaries. Ecological speciation scenarios are frequently invoked to explain such patterns, yet distinguishing between ecological adaptation and allopatric speciation remains challenging [95]. The dispersal of *P. erumei* from the Indian Ocean to the West Pacific Ocean may have been driven by the Equatorial Counter Current of the Indian Ocean gyre. Such ecological features greatly influence the evolution and adaptation of other flatfish species, including *Psettodes*, and have the potential to increase endemism in certain demersal ecosystems in marine settings. An intriguing case of rapid ecological speciation has been observed in Baltic flounder species, involving the development of a new ecological niche through a demersal spawning behavior, constituting the fastest speciation event documented for a marine vertebrate [32]. As a result, the peculiar spawning behavior attributes of *Psettodes* flatfishes, as well as ecological variables that may lead to reproductive isolation, suggest a process of ecological speciation.

### 3.7. Conservation Implication

Marine ecosystems remain relatively unexplored on a global scale, facing substantial gaps and challenges in terms of biodiversity assessment and the generation of genetic data [96,97]. In addition to the overarching influence of climate change, anthropogenic activities consistently pose threats to marine organisms, including flatfishes, across all oceanic regions [98,99,100]. Given the conservation significance of *Psettodes* flatfishes, the co-occurrence of both East Atlantic species within a portion of their range underscores the necessity for regulated harvesting within the framework of a multispecies fishery strategy. As global climate change progresses, marked alterations in environmental conditions such as elevated water temperatures, decreased oxygen levels, and modified wind patterns impact fish reproduction and recruitment on a global scale [101]. Consequently, increasing environmental variability is poised to exert negative repercussions on fish stock dynamics. Adaptations in fisheries and fishery management will likely encompass adjustments in fishing locales, target species, and the establishment of Marine Protected Areas (MPAs), highlighting the need for flexibility in both exploitation practices and management approaches. Nonetheless, predictions indicate that climate change will lead to heightened freshwater runoff in the eastern Atlantic Ocean (including basins of the Senegal, Gambia, Volta, Niger, and Congo Rivers), Indian Ocean (encompassing basins of the Zambezi, Limpopo, Indus, Ganges, Godavari, Brahmaputra, Irrawaddy, and Mekong Rivers), and western Pacific Ocean (such as the Pearl and Yangtze River Basins). This rise in runoff is anticipated to lower salinity levels, thereby constricting the distribution of numerous marine species. The reduction in salinity, driven by climate change, may exert severe selective pressure on pelagic-spawning species, potentially prompting a shift in reproductive behavior towards demersal spawning and even leading to local extinctions. Concurrently, the combined impact of eutrophication and climate change has accelerated the occurrence of hypoxia and anoxia in bottom waters where salinity conditions are conducive to pelagic spawning [102,103]. Consequently, the spawning habitat of pelagic flatfishes, currently delimited by geographic constraints, is likely to diminish in the future. This evolving scenario raises concerns about the potential local extinction of these species, underscoring the urgent need for comprehensive conservation strategies.

## 4. Conclusions

In conclusion, this study investigated the detailed analysis of the mitogenome of *P. belcheri*, shedding light on its genetic composition, structural organization, and evolutionary history. Furthermore, the genetic distances and phylogenetic relationships were explored, unveiling the evolutionary relationships among *Psettodes* species and their broader placement within the Pleuronectiformes order. Notably, the study identified significant genetic divergence between *P. belcheri* and *P. bennettii* in the mitochondrial COI gene, suggesting reproductive isolation despite their shared habitat. The phylogenetic relationship, divergence times, and diversification patterns were also estimated, providing insights into the emergence and distribution of *Psettodes* species in various oceanic regions. The interplay of hydrographic conditions, ocean currents, and ecological factors played crucial roles in shaping the evolutionary landscape of these flatfishes. Given the potential conservation implications, this study highlighted the need for a comprehensive understanding of marine ecosystems and the effects of climate change and anthropogenic activities on flatfish species. Conservation strategies and the establishment of MPAs were emphasized as essential components in safeguarding the diversity and sustainability of marine life, particularly species such as *Psettodes* flatfishes. In summary, the present analysis of the mitogenome and evolutionary history of *Psettodes* flatfishes offers valuable insights into the genetic attributes and possible ecological adaptations in East Atlantic and Indo-West Pacific Oceans.

## Figures and Tables

**Table 1:** List of annotated mitochondrial genes of *Psettodes belcheri*.

Genes	Start	End	Strand	Size (bp)	Intergenic Nucleotide	Anti-Codon	Start Codon	Stop Codon
tRNA-Phe	1	69	H	69	0	TTC	.	.
12S rRNA	70	1030	H	961	0	.	.	.
tRNA-Val	1031	1102	H	72	26	GTA	.	.
16S rRNA	1129	2830	H	1702	0	.	.	.
tRNA-Leu	2831	2903	H	73	0	TTA	.	.
ND1	2904	3878	H	975	4	.	ATG	AGG
tRNA-Ile	3883	3952	H	70	1	ATC	.	.
tRNA-Gln	3954	4024	L	71	−1	CAA	.	.
tRNA-Met	4024	4093	H	70	0	ATG	.	.
ND2	4094	5138	H	1045	0	.	ATG	T--
tRNA-Trp	5139	5211	H	73	2	TGA	.	.
tRNA-Ala	5214	5282	L	69	1	GCA	.	.
tRNA-Asn	5284	5356	L	73	38	AAC	.	.
tRNA-Cys	5395	5460	L	66	0	TGC	.	.
tRNA-Tyr	5461	5530	L	70	1	TAC	.	.
COI	5532	7082	H	1551	0	.	GTG	TAA
tRNA-Ser	7083	7153	L	71	8	TCA	.	.
tRNA-Asp	7162	7230	H	69	8	GAC	.	.
COII	7239	7929	H	691	0	.	ATG	T--
tRNA-Lys	7930	8004	H	75	1	AAA	.	.
ATP8	8006	8170	H	165	−7	.	ATG	TAA
ATP6	8164	8844	H	681	2	.	ATG	TA-
COIII	8847	9629	H	783	2	.	ATG	TA-
tRNA-Gly	9632	9702	H	71	0	GGA	.	.
ND3	9703	10,050	H	348	1	.	ATG	T--
tRNA-Arg	10,052	10,120	H	69	0	CGA	.	.
ND4L	10,121	10,414	H	294	−4	.	ATG	TAA
ND4	10,411	11,791	H	1381	0	.	ATG	T--
tRNA-His	11,792	11,859	H	68	0	CAC	.	.
tRNA-Ser	11,860	11,927	H	68	6	AGC	.	.
tRNA-Leu	11,934	12,006	H	73	0	CTA	.	.
ND5	12,007	13,845	H	1839	−1	.	ATG	TAA
ND6	13,845	14,363	L	519	0	.	ATG	TAG
tRNA-Glu	14,364	14,432	L	69	5	GAA		.
Cytb	14,438	15,578	H	1141	0	.	ATG	T--
tRNA-Thr	15,579	15,652	H	74	−1	ACA	.	.
tRNA-Pro	15,652	15,724	L	73	0	CCA	.	.
Control region	15,725	16,747	H	1023	.	.	.	.

**Table 2:** Nucleotide composition of the mitochondrial genomes of two *Psettodes* species.

Species Name	Size (bp)	A%	T%	G%	C%	A + T%	AT-Skew	GC-Skew
**Complete mitogenome**								
*P. belcheri* (OR231239)	16,747	28.09	26.06	16.24	29.56	54.15	0.037	−0.291
*P. erumei* (FJ606835)	17,315	28.83	24.78	15.71	30.68	53.61	0.076	−0.323
*P. erumei* (AP006835)	16,683	28.57	24.50	15.78	31.15	53.07	0.077	−0.328
**PCGs**								
*P. belcheri* (OR231239)	11,427	25.00	27.73	16.00	31.27	52.74	−0.052	−0.323
*P. erumei* (FJ606835)	11,427	25.37	25.75	15.62	33.25	51.13	−0.008	−0.361
*P. erumei* (AP006835)	11,426	25.33	25.62	15.62	33.43	50.95	−0.006	−0.363
**rRNAs**								
*P. belcheri* (OR231239)	2689	31.42	21.38	21.87	25.33	52.81	0.190	−0.073
*P. erumei* (FJ606835)	2680	32.43	20.56	20.86	26.16	52.99	0.224	−0.113
*P. erumei* (AP006835)	2680	32.43	20.45	20.90	26.23	52.87	0.227	−0.113
**tRNAs**								
*P. belcheri* (OR231239)	1556	27.83	27.31	23.14	21.72	55.14	0.009	0.032
*P. erumei* (FJ606835)	1553	27.17	26.85	23.82	22.15	54.02	0.006	0.036
*P. erumei* (AP006835)	1554	27.28	26.83	23.68	22.20	54.12	0.008	0.032
**CRs**								
*P. belcheri* (OR231239)	1015	30.25	33.40	12.51	23.84	63.65	−0.050	−0.312
*P. erumei* (FJ606835)	1601	38.91	33.92	10.31	16.86	72.83	0.069	−0.241
*P. erumei* (AP006835)	968	42.15	36.36	7.13	14.36	78.51	0.074	−0.337

## Data Availability

The genome sequence data that support the findings of this study are openly available in GenBank of NCBI at https://www.ncbi.nlm.nih.gov, accessed on 15 August 2023, under the accession no. OR231239.

## Supplementary Materials

The following supporting information can be downloaded at: https://www.mdpi.com/article/10.3390/biology12101317/s1, Table S1. Dataset of Pleuronectiformes flatfishes for present phylogenetic analyses (GenBank Accessed on 15 August 2023); Table S2. Comparison of the intergenic nucleotides of two *Psettodes* species mitogenomes.; Table S3. Start and stop codons of all 13 PCGS in two *Psettodes* species mitogenomes; Figure S1. Mitochondrial COI-based Bayesian phylogeny clearly discriminate three *Psettodes* species with high posterior probabilities branch supports. The K2P genetic distances of three *Psettodes* species were overlaid on the topology.
