# Supplementary material for: Insights into the Mitochondrial Genetic Makeup and Miocene Colonization of Primitive Flatfishes (Pleuronectiformes: Psettodidae) in the East Atlantic and Indo-West Pacific Ocean
Source: Biology (Basel). 2023 Oct 9;12(10):1317. doi: 10.3390/biology12101317 (PMC10604034; doi:10.3390/biology12101317)
Supplement: Supplementary file 1 [file biology-12-01317-s001.zip › Figure S1.pdf]

Figure S1. Mitochondrial COI based Bayesian phylogeny clearly discriminate three *Psettodes* species with high posterior probabilities branch supports. The K2P genetic distances of three *Psettodes* species were overlaid on the topology.

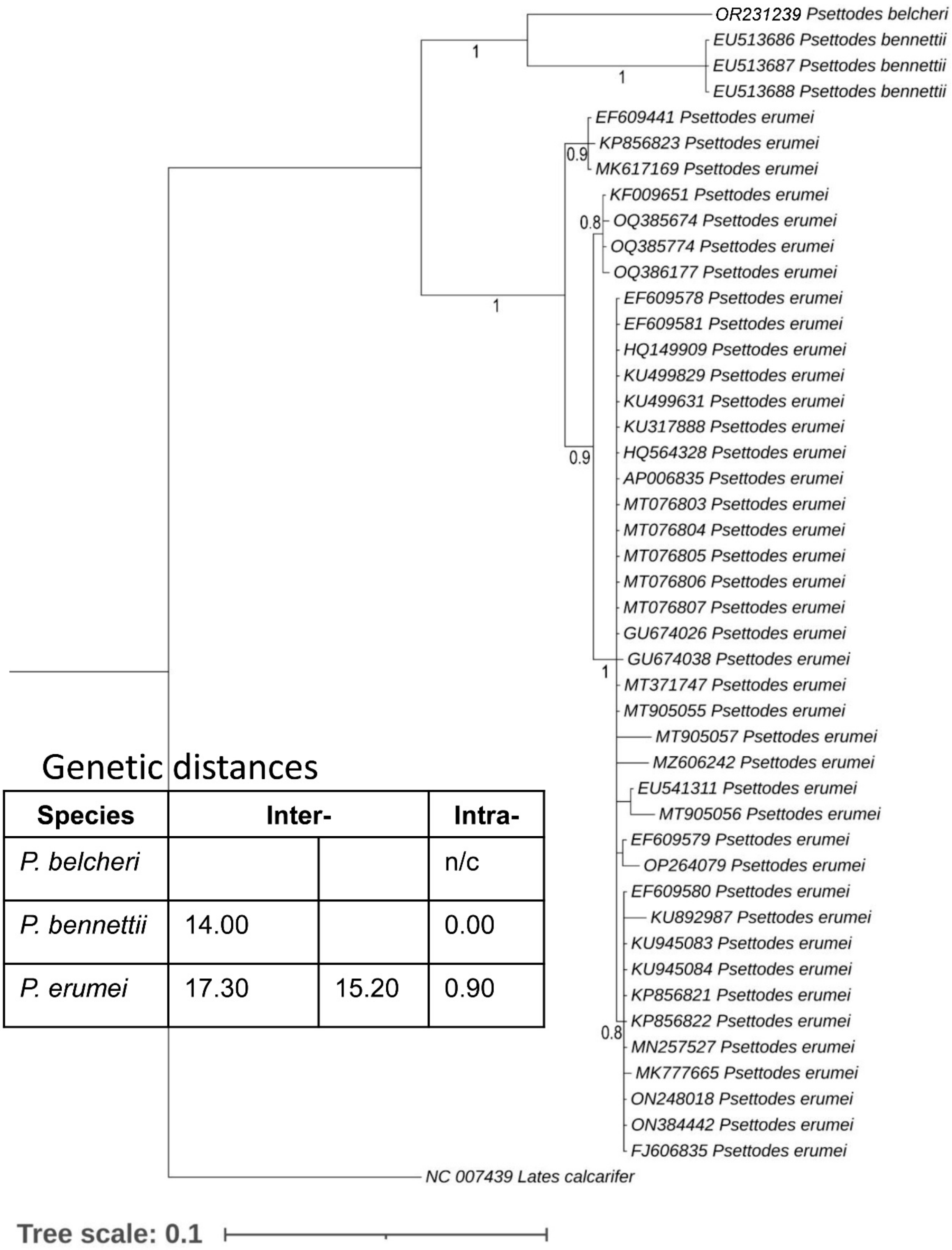

Species	Inter-		Intra-
P. belcheri			n/c
P. bennettii	14.00		0.00
P. erumei	17.30	15.20	0.90
