# Supplementary material for: Insights into the Mitochondrial Genetic Makeup and Miocene Colonization of Primitive Flatfishes (Pleuronectiformes: Psettodidae) in the East Atlantic and Indo-West Pacific Ocean
Source: Biology (Basel). 2023 Oct 9;12(10):1317. doi: 10.3390/biology12101317 (PMC10604034; doi:10.3390/biology12101317)
Supplement: Supplementary file 1 [file biology-12-01317-s001.zip › Table S1.pdf]

Table S1. Dataset of Pleuronectiformes flatfishes for present phylogenetic analyses.

Sl. No.	Sub Order	Family	Species Name	Accession Number	References
1	Psettodoidei	Psettodidae	*Psettodes belcheri*	OR231239	This study
2			*Psettodes erumei*	FJ606835	GenBank
3			*Psettodes erumei*	AP006835	GenBank
4	Pleuronectoidei	Achiridae	*Trinectes maculatus*	JQ639070	GenBank
5		Bothidae	*Bothus myriaster*	KJ433563	[47]
6		Citharidae	*Lepidoblepharon ophthalmolepis*	AP014592	[28]
7		Cyclopsettidae	*Cyclopsetta fimbriata*	AP014590	[28]
8		Cynoglossidae	*Symphurus orientalis*	KP992899	[45]
9			*Cynoglossus gracilis*	KT809367	[48]
10		Paralichthyidae	*Pseudorhombus dupliciocellatus*	KJ433562	[49]
11		Pleuronectidae	*Verasper moseri*	EF025506	[43]
12		Rhombosoleidae	*Neoachiropsetta milfordi*	AP014593	[28]
13		Samaridae	*Samariscus latus*	KF494223	[44]
14		Scophthalmidae	*Scophthalmus maximus*	EU419747	GenBank
15		Soleidae	*Zebrias crossolepis*	KJ433564	[46]
16		Centropomidae	*Lates calcarifer* (out group)	DQ010541	[42]
