# Supplementary material for: Insights into the Mitochondrial Genetic Makeup and Miocene Colonization of Primitive Flatfishes (Pleuronectiformes: Psettodidae) in the East Atlantic and Indo-West Pacific Ocean
Source: Biology (Basel). 2023 Oct 9;12(10):1317. doi: 10.3390/biology12101317 (PMC10604034; doi:10.3390/biology12101317)
Supplement: Supplementary file 1 [file biology-12-01317-s001.zip › Table S2.pdf]

Table S2. Comparison of the intergenic nucleotides of two *Psettodes* species mitogenomes.

Genes	*Psettodes belcheri* (OR231239)	*Psettodes erumei* (FJ606835)	*Psettodes erumei* (AP006835)
tRNA-Phe	0	0	0
12S rRNA	0	0	0
tRNA-Val	26	0	0
16S rRNA	0	0	0
tRNA-Leu	0	0	0
ND1	4	4	4
tRNA-Ile	1	3	3
tRNA-Gln	-1	-1	-1
tRNA-Met	0	0	0
ND2	0	0	0
tRNA-Trp	2	1	1
tRNA-Ala	1	1	1
tRNA-Asn	38	37	38
tRNA-Cys	0	0	0
tRNA-Tyr	1	1	1
COI	0	0	0
tRNA-Ser	8	10	10
tRNA-Asp	8	8	8
COII	0	0	0
tRNA-Lys	1	1	1
ATP8	-7	-10	-10
ATP6	2	0	0
COIII	2	0	0
tRNA-Gly	0	0	0
ND3	1	0	0
tRNA-Arg	0	0	0
ND4L	-4	-7	-7
ND4	0	0	0
tRNA-His	0	0	0
tRNA-Ser	6	6	6
tRNA-Leu	0	0	0
ND5	-1	-4	-4
ND6	0	0	0
tRNA-Glu	5	5	5
Cytb	0	0	0
tRNA-Thr	-1	-1	-1
tRNA-Pro	0	0	0
CR			
