# Supplementary material for: Insights into the Mitochondrial Genetic Makeup and Miocene Colonization of Primitive Flatfishes (Pleuronectiformes: Psettodidae) in the East Atlantic and Indo-West Pacific Ocean
Source: Biology (Basel). 2023 Oct 9;12(10):1317. doi: 10.3390/biology12101317 (PMC10604034; doi:10.3390/biology12101317)
Supplement: Supplementary file 1 [file biology-12-01317-s001.zip › Table S3.pdf]

Table S3. Start and stop codons of all 13 PCGS in two *Psettodes* species mitogenomes.

Genes	*Psettodes belcheri* (OR231239)		*Psettodes erumei* (FJ606835)		*Psettodes erumei* (AP006835)	
	Start	Stop	Start	Stop	Start	Stop
ND1	ATG	AGG	ATG	AGG	ATG	AGG
ND2	ATG	T--	ATG	T--	ATG	T--
COI	GTG	TAA	GTG	TAA	GTG	TAA
COII	ATG	T--	ATG	T--	ATG	T--
ATP8	ATG	TAA	ATG	TAA	ATG	TAA
ATP6	ATG	TA-	ATG	TA-	ATG	TA-
COIII	ATG	TA-	ATG	TA-	ATG	TA-
ND3	ATG	T--	ATG	T--	ATG	T--
ND4L	ATG	TAA	ATG	TAA	ATG	TAA
ND4	ATG	T--	ATG	T--	ATG	T--
ND5	ATG	TAA	ATG	TAA	ATG	TAA
ND6	ATG	TAG	ATG	TAG	ATG	TAG
Cytb	ATG	T--	ATG	T--	ATG	T--
